# Evaluation of Awareness, Knowledge, and Attitude Toward Basic Life Support Among the General Population in Saudi Arabia: A Nationwide Survey

**DOI:** 10.7759/cureus.71214

**Published:** 2024-10-10

**Authors:** Bader Alghamdi, Faisal F Alshehri, Buthaina M Alsharif, Sara K Habib, Malak K AlSugayer, Noora A Juaythin, Watin A Aldrebi

**Affiliations:** 1 Department of Emergency Medicine, Prince Sultan Military Medical City, Riyadh, SAU; 2 College of Medicine, Imam Mohammad Ibn Saud Islamic University (IMSIU), Riyadh, SAU

**Keywords:** attitude, awareness, basic life support, knowledge, saudi arabia

## Abstract

Background

Basic life support (BLS) is the key component in changing the outcome from death to survival when cardiac arrest occurs. It involves providing cardiopulmonary resuscitation (CPR) and utilizing an automated external defibrillator (AED) to help restore the normal cardiac rhythm. By equipping more individuals with BLS training, it will enhance community preparedness and public health.

Aim

This study aimed to evaluate the general population's awareness, knowledge, and attitude toward BLS in Saudi Arabia.

Subject and methods

This cross-sectional study was conducted among the Saudi general population covering all five main regions in Saudi Arabia (Central, Eastern, Western, Southern, and Northern). A self-administered questionnaire was sent through volunteer data collectors in every area in which they live to the population using a Google (Google, Inc., Mountain View, CA) survey. The questionnaire includes socio-demographic characteristics (i.e., age, gender, region of residence, etc.) to assess the awareness, knowledge, and attitude toward BLS using a 20-item questionnaire based on the American Heart Association (AHA).

Results

Of the 992 participants, 615 (62%) were females, and 325 were aged between 26 and 35 years (32.8%). Four hundred eleven participants (41.4%) attended training related to BLS. The most common source of training information was a resuscitation society course (51.8%), followed by the university curriculum (24.6%) and school (10.7%). The overall mean knowledge score was 9.64 (SD 2.92) out of 20 points. Among them, 487 (49.1%) had moderate knowledge levels, 466 (47%) were poor, and only 39 (3.9%) had good knowledge. Factors associated with increased knowledge include younger age, living outside Central Region, better education, being a healthcare worker, knowing how to give a cardiac massage, previous participation in BLS training, and witnessed of sudden death. The biggest barrier that would prevent participants from providing a cardiac massage is fear of making a mistake (63.1%).

Conclusion

The general population's knowledge of BLS was found to be inadequate. However, younger participants who had a better education and worked in a healthcare institution tended to be more knowledgeable about BLS than the rest of the groups. The gaps in the knowledge are evidently seen in this study. Hence, appropriate measures are needed to bridge knowledge gaps. Healthcare authorities should devise a program to deliver necessary information about the basic facts of BLS throughout the community.

## Introduction

Cardiac arrest remains one of the leading causes of sudden death among individuals globally, including in Saudi Arabia [1]. According to the American Heart Association (AHA), around 90% of outside-hospital cardiac arrest incidents happen at home or in public places [2]. Basic life support (BLS) is an immediate life-saving measure provided by the healthcare professional or a trained first responder to deliberately increase the survival chance of a person suffering from cardiac arrest through the delivery of cardiopulmonary resuscitation (CPR) and the use of automated external defibrillators (AED). Early application of BLS techniques can significantly impact the outcome of cardiac arrest and could potentially improve mortality among the affected persons [3].

Awareness of the general public about life-saving measures such as BLS and CPR can make a huge difference during events. Amid the cardiac arrest crisis, time is very crucial to the victim’s survivability. Every minute delays may result in irreversible organ damage or death. Having someone around who has a background in BLS principles, whether a healthcare provider or a bystander, can inadvertently improve the situation and save the life of the person in cardiac arrest. BLS-trained non-healthcare individuals in the population can provide immediate and temporary life-saving support until a professional emergency response is available and the person is brought to the hospital [4]. Various methods can be used to improve public knowledge of BLS concepts. The use of multimedia platforms and community training programs, such as mandatory courses in schools and workplaces, are some of the effective strategies for increasing BLS awareness [5].

Knowledge and awareness of the general public on BLS

Numerous studies have tackled the general public knowledge and awareness of BLS in Saudi Arabia. In an article by Khashaba et al. (2021), around 84% of non-healthcare providers in Riyadh stated they had limited knowledge of BLS concepts [6]. The same findings were concluded by Subki et al. (2018) in a similar study conducted in Jeddah [7]. A cross-sectional study among students from different schools and universities across Saudi Arabian regions also found that most participants reported inadequate knowledge of BLS and CPR [8-11]. Interestingly, Asiri et al. (2020) discussed that Saudi males demonstrated significantly better knowledge and CPR skills as compared with female Saudi counterparts [12]. The same results were identified in other parts of the world, such as Pakistan, China, and New Zealand, where the general public's knowledge of BLS and CPR is low [13-15].

The proportion of BLS trained among the general population in Saudi Arabia was 22% in Riyadh and 19.8% in Al Qassim [6,16]. In a systematic review and meta-analysis conducted by Alsabri et al. (2024) to determine the BLS skill level and training among non-healthcare providers in Arab countries, 55% stated they had awareness, but only 28% were confident and had sufficient knowledge of providing BLS [17]. As compared with other published literature from different countries, a study in Turkey reported that 40.7% had previous BLS training, while 17.8% in Portugal, 11% in China, and only 8.9% in Ethiopia [14,18-20].

The attitude of the general public toward BLS

Most Saudi Arabian survey participants demonstrated a positive attitude toward the importance of BLS. University students are convinced that incorporating BLS courses into their curriculum could potentially improve their skills and awareness [9,11]. A study in Hong Kong discussed possible reasons why BLS and CPR skills among non-healthcare providers are inadequate. Participants expressed a lack of interest and felt that acquiring BLS was unnecessary for their social responsibilities. However, individuals who attended higher education demonstrate more willingness to participate in BLS training programs [21]. A study in China explores whether BLS-trained bystanders will be willing to help a person who is in cardiac arrest. Surprisingly, participants would be more likely to perform CPR if it ever happened to their family members, and fewer would be willing to perform it on strangers. Participants voiced concerns over legal implications, such as fear of being prosecuted if CPR was unsuccessful [22]. Similar findings were found in a study in Jordan, wherein the majority of the participants would prefer to call the ambulance rather than perform BLS or chest compression and mouth-to-mouth resuscitation to strangers if they were bystanders in the event of cardiac arrest [23].

To address these gaps, the majority of researchers agreed that increasing awareness and improving the knowledge of the community regarding BLS may significantly improve the mortality and survival rate of any person suffering from cardiac arrest. Most of these studies suggest that enhancing the skills of the general population in BLS and CPR techniques can be achieved through mandatory community training by conducting courses using different channels such as the workplace, school, and multimedia platforms [23-25]. Furthermore, the role of governments could also make a difference by passing a law that will protect bystanders when BLS and CPR are essentially needed [22-23].

The literature demonstrates that acquiring BLS skills is critical for the general public since it can substantially improve cardiac arrest outcomes during emergencies. To our knowledge, a review of research completed in Saudi Arabia demonstrated that few investigations were conducted to assess the public's understanding of BLS, with no single study addressing the whole nation's regions. Further research, particularly at the national level, must identify effective techniques for increasing BLS training enrollment and eliminating barriers to bystander response. As a result, this study intends to assess the Saudi general population's awareness, knowledge, and attitude toward BLS.

## Materials and methods

Study design

This was an analytical cross-sectional study that included the general population of Saudi Arabia for all five regions in the country, which are Central, Eastern, Western, Southern, and Northern, between May 2023 and April 2024. The study is authorized by King Faisal University's Institutional Review Board (IRB). The study's aims were explained to all volunteers, and their consent was obtained by noting that their continued participation and submission of the survey were considered acceptance. All responses from this study were kept completely confidential, with only the study's authors having full access for research purposes only, and all information provided by volunteers was treated with the utmost confidentiality.

Study subjects and size

Participants in this study were members of the general public whose education ranged between all different academic levels, including healthcare workers and non-healthcare workers in Saudi Arabia. All participants must meet the following inclusion criteria: those who live in Saudi Arabia and are 16 years and older. The minimum sample size needed for a population of Saudi Arabia, estimated at 20,000, to achieve a precision of ±5% with a 95% confidence interval, was calculated to be 385 participants, considering the estimated proportions of healthcare workers (20%) and non-healthcare workers (80%). After the data collection period, the total sample size was increased to 992 participants to ensure better representation and accommodate potential non-responses.

Application of the questionnaire

The survey was divided into two sections. The first section addresses the independent variables: the participants' demographic and personal data, such as gender, age, nationality, region, education level, and occupation, such as whether they work inside or outside the healthcare field. The second section focused on the dependent variables by evaluating the participants' awareness, knowledge, and attitude toward BLS. The questionnaire was developed based on the AHA guidelines. The questionnaire was written in two languages: English and Arabic. It includes a total of 20 questions that are divided into eight main themes, namely, CPR/BLS training experience, clinical features of cardiac arrest, participants' perception toward CPR, indications of initiating CPR, techniques of chest compression, recognition of AED/defibrillation, participants' action toward arrested victims (including family members, friends, and strangers), Participants' self-reflection on CPR (during and after). However, while there is no specific validation of the questionnaire in the context of the Saudi population, it is important to note that the tool has been successfully used in Jordan, a nearby country that shares similar cultural, educational, and linguistic contexts. This similarity provides a reasonable basis for believing that the questionnaire will perform accurately and reliably within the Saudi population. [23].

Sampling technique

For data collection, a non-probability convenience sampling technique is used. Data collectors in all five regions are recruited voluntarily by a post published on the Telegram app (Telegram Messenger LLP, London, UK) for the opportunity of data collection to gather data from the population in their area via an online self-administered survey distributed through social media platforms (WhatsApp (Meta, Menlo Park, CA), Telegram, X (X Corp., San Francisco, CA), Facebook (Meta, Menlo Park, CA), etc.), their acquaintances, or random public face-to-face.

Questionnaire criteria

The knowledge toward BLS has been assessed using a 20-item questionnaire, with the correct answer for each question identified and coded with 1, while the incorrect answer was coded with 0. The total knowledge score was calculated by adding all 20 items. A possible score ranging from 0 to 20 points was achieved; the higher the score, the higher the knowledge of BLS. By using 50% and 75% as cutoff points to determine the level of knowledge, respondents were considered poor if the score was less than 50%, 50% to 75% were considered moderate, and above 75% were considered as having good knowledge levels.

Statistical analysis

The data were analyzed using the software program Statistical Packages for Software Sciences (SPSS) version 26 (IBM SPSS Statistics for Windows, IBM Corp., Armonk, NY). Descriptive statistics were given as numbers and percentages (%) for all categorical variables, while continuous variables were calculated and summarized as mean and standard deviation. The association between the knowledge score among the socio-demographic characteristics and previous experiences of BLS was evaluated using the Mann-Whitney Z-test and Kruskal-Wallis H-test. The normality test (statistical collinearity) was performed using the Shapiro-Wilk test as well as the Kolmogorov-Smirnov test. According to the results, the knowledge scores follow a non-normal distribution. Thus, the non-parametric test was applied. Values were considered significant with a p-value of less than 0.05.

## Results

In total, 992 participants completed the survey. As described in Table 1, 325 (32.8%) were aged between 26 and 35 years old, 615 with the majority being females (62%). Nearly all 940 (94.8%) were Saudis, and approximately 479 (48.3%) lived in the Central Region. Respondents who were bachelor's degree holders constituted 63.6%. About three-quarters of 717 (72.3%) were non-healthcare workers. The proportion of participants who knew how to give cardiac massage in case of sudden death was 396 (39.9%). In addition, 411 (41.4%) had previous participation in training related to BLS.

**Table 1:** Socio-demographic characteristics of participants (n = 992)

Study data	n (%)
Age group	
16-25 years	318 (32.1%)
26-35 years	325 (32.8%)
36-45 years	115 (11.6%)
46-55 years	129 (13.0%)
>55 years	105 (10.6%)
Sex	
Male	377 (38.0%)
Female	615 (62.0%)
Nationality	
Saudi	940 (94.8%)
Non-Saudi	52 (05.2%)
Residence region	
Central Region	479 (48.3%)
Eastern Region	188 (19.0%)
Western Region	105 (10.6%)
Northern Region	129 (13.0%)
Southern Region	91 (09.2%)
Educational level	
Primary school	03 (0.30%)
Middle school	14 (01.4%)
High school	136 (13.7%)
Diploma/higher diploma degree	79 (08.0%)
Bachelor degree	631 (63.6%)
Postgraduate	129 (13.0%)
Occupation	
Healthcare worker	275 (27.7%)
Non-healthcare worker	717 (72.3%)
Do you know how to give cardiac massage in the case of cardiac arrest and respiratory standstill (sudden death)?	
Yes	396 (39.9%)
No	596 (60.1%)
Previous participation in training related to basic life support (BLS)	
Yes	411 (41.4%)
No	581 (58.6%)

**Figure 1:**
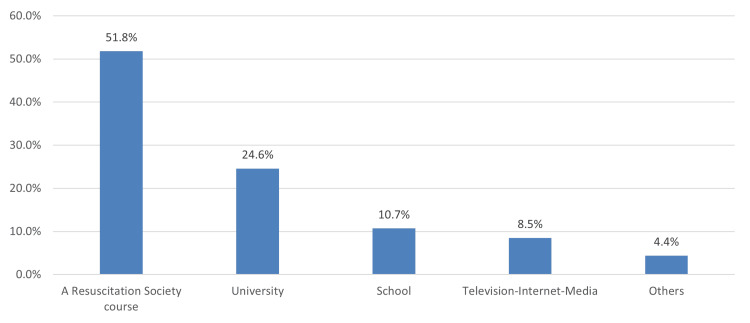
Sources of basic life support (BLS) information

In Figure 1, among those who received previous training related to BLS (411, 41.4%), the most common source of training information was a resuscitation society course (514, 51.8%), followed by the university curriculum (244, 24.6%) and school (106, 10.7%).

**Figure 2:**
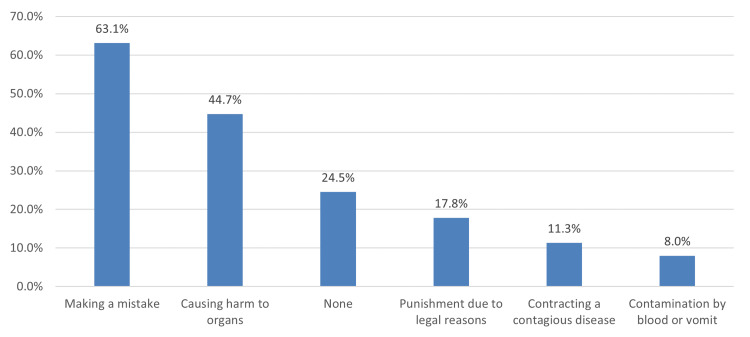
Barriers to providing cardiac massage

The most common barrier to providing cardiac massage was fear of making a mistake (626, 63.1%), followed by causing harm to organs (443, 44.7%) and punishment due to legal reasons (177, 17.8%) (Figure 2).

In Table 2, the prevalence of participants who previously witnessed sudden death was 137 (13.8%). Of them, the most common action was calling the ambulance (78, 56.9%). When asked if, by chance, they encountered a person whose heart had stopped, the most common action to be taken using the BLS application was to do both ventilation and cardiac massage (501, 50.5%). The proportion of respondents who knew the location of the automated defibrillator or pacemaker was 431 (43.4%). When asked what action to take if someone from a family member or friend fainted/unconscious, the most common action to be taken was to call an ambulance (611, 61.6%). When asked if witnessing sudden death in a stranger, the most common action was also to call an ambulance (695, 70.1%).

**Table 2:** Assessment of attitude toward basic life support (BLS) † Number of participants who ever witnessed sudden death CPR, cardiopulmonary resuscitation

Statement	n (%)
Have you ever witnessed sudden death?	
Yes	137 (13.8%)
No	855 (86.2%)
What did you do in this situation? ^(n = 137)^^†^	
I began to give a cardiac massage.	41 (29.9%)
I conducted mouth-to-mouth ventilation (I respirated).	05 (03.6%)
I gave a cardiac massage and conducted mouth-to-mouth ventilation (I gave CPR).	40 (29.2%)
I called an ambulance.	78 (56.9%)
I told somebody to call for help.	64 (46.7%)
I just watched and left.	18 (13.1%)
If you are confronted with a person whose heart has stopped, which of the basic life support applications can you apply?	
I can open the airway and ventilate/conduct mouth-to-mouth ventilation (kiss of life).	91 (09.2%)
I can give a cardiac massage.	178 (17.9%)
I can both ventilate and give a cardiac massage.	501 (50.5%)
I do not know.	222 (22.4%)
Do you have any idea about where an "automated external defibrillator" or "pacemaker" can be found?	
Yes.	431 (43.4%)
I do not know.	561 (56.6%)
If someone from your family members or friends fainted/unconscious (sudden death), what would you do?	
I would begin to give a cardiac massage.	271 (27.3%)
I would call an ambulance.	611 (61.6%)
I would call somebody or call for help.	36 (03.6%)
I do not know what to do.	74 (07.5%)
What would you do if you witnessed a stranger fainted/unconscious (sudden death)?	
I would begin to give a cardiac massage.	202 (20.4%)
I would call an ambulance.	695 (70.1%)
I would call somebody or call for help.	22 (02.2%)
I do not know what to do.	73 (07.4%)

Regarding the assessment of the knowledge of BLS (Table 3), only 95 (9.6%) were correct that loss of consciousness, discontinuation of breathing and/or circulation, and cyanosis were the indications of sudden cardiac arrest. Approximately 629 (63.4%) of respondents were aware that the consciousness state of a person can be determined when there is no response when called. In the absence of respiration, 526 (53%) indicated that it can be determined if there is no respiratory movement. For the absence of circulation, 527 (53.1%) were correct that it can be determined when not feeling a pulse in the neck vessels (53.1%). Approximately 409 (41.2%) knew the correct meaning of cardiac massage. However, poor knowledge was seen in the proper rate of cardiac massage/artificial ventilation during cardiac massage either in "one" rescuer or "two" rescuers. Nearly half (475, 47.9%) knew that the middle of the chest was the correct area to apply cardiac massage. Only 157 (15.8%) knew the rate of cardiac massage, and only 329 (33.2%) knew the force that must be applied during heart massage. In addition, 767 (77.3%) were aware of the correct meaning of defibrillator. The overall mean knowledge score was 9.64 (SD 2.92), with poor, moderate, and good knowledge levels constituting 466 (47%), 487 (49.1%), and 39 (3.9%), respectively.

**Table 3:** Assessment of knowledge toward basic life support (BLS)

Statement	n (%)
Indication of sudden cardiac arrest (loss of consciousness, discontinuation of breathing and/or circulation, and cyanosis)	95 (09.6%)
How can the consciousness state of the individual be determined?	
No response when called (yes)	629 (63.4%)
No response when touched (yes)	525 (52.9%)
Not moving at all (yes)	444 (44.8%)
How can the absence of respiration be determined?	
Not having any respiratory movement (yes)	526 (53.0%)
Not having any respiratory sound (yes)	456 (46.0%)
Not coming air out of the mouth of the individual (yes)	358 (36.1%)
Not steaming up a mirror placed in front of the mouth of the individual (no)	646 (65.1%)
How can the absence of circulation be determined?	
The lack of circulation signs (no)	460 (46.4%)
Not feeling a pulse in the vessels of the neck (yes)	527 (53.1%)
Not feeling a pulse in the vessels of the arm (no)	613 (61.8%)
What do you think a "cardiac massage" means? (to apply strong compression to the chest at certain intervals (compress))	409 (41.2%)
What is the proper rate of cardiac massage/artificial ventilation during cardiac massage if there is "one" rescuer? (30/2)	243 (24.5%)
What is the proper rate of cardiac massage/artificial ventilation during cardiac massage if there are "two" rescuers? (30/2)	138 (13.9%)
Which area must cardiac massage be applied on? (middle of the chest)	475 (47.9%)
What must be the rate of the cardiac massage? (at least 100 times per minute)	157 (15.8%)
How much force must be applied during heart massage? (moderate force, such that the rib cage moves down 5 to 6 cm)	329 (33.2%)
What do you know about the device defined as a "defibrillator" that is used during cardiac massage when necessary?	
It is a device supporting respiration (no)	909 (91.6%)
It is a device to restart a heart that has stopped working (yes)	767 (77.3%)
I have never heard of it (no)	785 (79.1%)
Total knowledge score (mean ± SD)	9.64 ± 2.92
Level of knowledge	
Poor	466 (47.0%)
Moderate	487 (49.1%)
Good	39 (03.9%)

When measuring the association between the knowledge score in terms of the socio-demographic characteristics and previous experience of sudden death (Table 4), it was observed that a higher knowledge score was more associated with being younger (Z = 5.869; p < 0.001), living outside Central Region (Z = 2.001; p = 0.045), respondents with bachelor or higher degree holders (Z = 3.014; p = 0.003), being a healthcare worker (Z = 13.242; p < 0.001), knew how to give cardiac massage (Z = 16.328; p < 0.001), previous participation in training related to BLS (Z = 14.922; p < 0.001), and witnessed sudden death (Z = 5.194; p < 0.001).

**Table 4:** Association between the knowledge score and the socio-demographic characteristics of participants and previous witnesses of sudden death § p-value has been calculated using Mann Whitney Z-test. ** Significant at p < 0.05 level.

Factor	Knowledge score (20), mean ± SD	Z-test	p-value ^§^
Age group			
≤35 years	10.0 ± 2.89	5.869	<0.001 **
>35 years	8.90 ± 2.83		
Gender			
Male	9.69 ± 3.11	0.716	0.474
Female	9.61 ± 2.79		
Nationality			
Saudi	9.66 ± 2.89	0.661	0.509
Non-Saudi	9.31 ± 3.31		
Residence region			
Inside Central Region	9.48 ± 2.67	2.001	0.045 **
Outside Central Region	9.79 ± 3.12		
Educational level			
Diploma or below	9.09 ± 2.82	3.014	0.003 **
Bachelor or higher	9.81 ± 2.93		
Occupation			
Healthcare worker	11.6 ± 2.76	13.242	<0.001 **
Non-healthcare worker	8.87 ± 2.59		
Do you know how to give cardiac massage in the case of cardiac arrest and respiratory standstill (sudden death)?			
Yes	11.5 ± 2.42	16.328	<0.001 **
No	8.41 ± 2.56		
Previous participation in training related to basic life support (BLS)			
Yes	11.3 ± 2.53	14.922	<0.001 **
No	8.48 ± 2.60		
Witnessed sudden death			
Yes	10.8 ± 2.87	5.194	<0.001 **
No	9.45 ± 2.88		

## Discussion

This study investigated the general population's knowledge about BLS and determined their attitude when encountering a case of cardiac arrest. In this study, the population's knowledge of BLS was insufficient. Based on our criteria, out of 20 knowledge items, the overall mean knowledge score was 9.64 (SD 2.92). Accordingly, we observed that nearly half of our respondents, 466 (47%), were considered to have a poor level of knowledge, 487 (49.1%) were moderate, and fewer than 39 (4%) were considered to have good knowledge levels. Consistent with our findings, several studies documented a lack of knowledge about BLS, whether the general population group [15,16,19,12,23], non-healthcare providers group [6,7], students group [9-11], or even healthcare providers group [13]. Contradicting these reports, a study conducted in Ethiopia [20] reported better BLS knowledge among the nonmedical population group, as nearly half of 183 (44.4%) were knowledgeable about BLS, which was consistent with the study done in Jazan [8]. The differences in the level of BLS knowledge could be due to varying reasons, including the number of sample populations, population diversity, regional settings, etc. Improving knowledge is always a challenge among authorities. Hence, collective efforts are crucial to achieving better information dissemination throughout the community.

Data from our study indicate that demographic factors influencing the level of knowledge include younger age, living outside the Central Region, having a bachelor's or higher degree, and being a healthcare worker. This is almost consistent with the study of Teng et al. (2020) [14]. The study identified younger participants working in healthcare institutions, and those who had better education achieved a better rate of CPR training. This is corroborated by the paper of Khashaba et al. (2021) [6], which reports a significant association between the level of knowledge according to gender, education, and occupation; however, in a study done by Saquib et al. (2019) [11], they found that female participants, as well as medical interns, were more associated with higher scores in awareness. These were not observed in our study, as we found no significant differences between the knowledge score in terms of gender and nationality (p > 0.05), which was consistent with the study of Alghamdi et al. (2021) [25]. Furthermore, each of these studies focused on different parameters, reflecting a positive outcome at the end.

Incidentally, our results revealed that witnessing sudden death, previous participation in BLS training, and knowledge of performing cardiac massage were the most significant factors in BLS knowledge. Across publications, prior BLS-related training was proven to be the most substantial contributor to knowledge [13,20,16,19]. Furthermore, we noted that a little below half of our population had previously attended BLS training, with a resuscitation society course (514, 51.8%), university curriculum (244, 24.6%), and school (106, 10.7%) being the most common source of BLS training information. Among the Turkish sample population who attended CPR training [18], the most common site of training was the workplace and school. This mirrored the results of a study conducted in Ireland [24], where trained individuals reported the workplace as their primary source of information for both awareness and training, while untrained persons cited a lack of knowledge regarding the necessity of CPR training as their primary reason for being untrained.

Regarding the specific assessment of the knowledge, our results showed that although the majority of our respondents seemed to have mediocre knowledge on how to determine consciousness state, absence of respiration, and absence of circulation, significant gaps were seen in the correct indications of cardiac arrest, the exact rate of cardiac massage/artificial ventilation either for "one" or "two" rescuers. Also, few of them knew about the rate of cardiac massage and the mandatory force that should be applied. However, despite this scenario, most of our respondents knew the correct meaning of the defibrillator. In New Zealand [15], the community's knowledge of the chest compression rate and the correct compression-to-ventilation ratio was relatively low. On the contrary, in Turkey [18], the general public exhibited better awareness of CPR. Approximately half of the population indicated the loss of consciousness, cessation of breathing, and cessation of circulation as the most common signs of cardiac arrest. Also, 277 (52%), 182 (34.3%), and 83 (15.6%) were aware of the location for performing chest compression, correct depth, and compression ventilation rate, respectively. Below half of our population had previously attended BLS training; with a resuscitation society course, university curriculum, and school, it is a very important and successful way to gain BLS information and train for it.

It is important to discuss the attitude of our respondents toward sudden death. For instance, more than half of our population would call an ambulance for the occurrence of sudden death, whether family members, friends, or strangers. In the case of BLS applications, half of our respondents were more likely to perform both cardiac massage and mouth-to-mouth ventilation. The attitude of the general public in China toward CPR seems to be better than our results [22]. Nearly all subjects would perform CPR on their family members, although the rate slightly decreased for strangers. This mirrored the results in Jeddah [25], as most respondents would perform CPR without hesitation if the incident involved their family members, which represents a strong factor.

The barriers to providing cardiac massage are detrimental factors for knowledge. In our study, fear of making a mistake, fear of causing harm to organs, and fear of punishment due to legal reasons were some of the most prominent reasons for not providing CPR. These results are comparable to the study of Jarrah et al. (2018) [23], wherein fear of committing mistakes was the major reason for students not performing CPR, which was also consistent with the reports of Özbilgin et al. (2015) [18]. These barriers are the roadblocks to improving the population's knowledge; hence, efforts should be made to address the effects of these barriers.

Our research has limitations that should be noted. Cross-sectional studies utilizing convenient methods of sampling that rely on online self-reported data have an increased risk of bias. However, our study utilized an AHA-based questionnaire to assess Saudi society's knowledge and attitude. Even if our research focused on knowledge, awareness, and attitude, the instrument should be examined for reliability to ensure that it regularly delivers similar findings when given to the Saudi population. Reaching a larger sample proved challenging due to the lack of recognition of the importance of surveys in our society. The general population of Saudi Arabia may misreport their knowledge due to variables such as education, prejudice, or fear. Furthermore, the convenience sampling method has limited representativeness and may lack diversity in the study population. Also, because the data are chosen based on availability, it may lead to overrepresentation of certain groups or opinions, skewing the results. In addition, the non-parametric distribution of data tends to have less precision of population estimation parameters and may not give as much information about the associations between variables as parametric tests.

## Conclusions

There was unsatisfactory knowledge among the general population regarding BLS. However, younger participants with better education and healthcare workers tended to be more knowledgeable about BLS than the rest of the population. In case of sudden, our population was more likely to call an ambulance rather than give a cardiac massage. This could be due to the fear of making mistakes and causing harm to organs. Further, knowledge of giving a cardiac massage, previous participation in BLS training, and witnessing sudden death were identified as the most important contributors to the general population's knowledge about BLS. However, given this scenario, the general public needs to increase their knowledge of BLS. Devising new policies to boost the knowledge of the population, particularly the older population group, those who were less educated and non-healthcare workers, could result in better perspectives from the public regarding BLS. In addition, awareness campaigns, particularly through social media and the provision of a BLS course, could definitely increase the community's knowledge and attitude toward sudden illness.
